# Unprocessed Viral DNA Could Be the Primary Target of the HIV-1 Integrase Inhibitor Raltegravir

**DOI:** 10.1371/journal.pone.0040223

**Published:** 2012-07-02

**Authors:** Farah F. Ammar, Safwat Abdel-Azeim, Loussinée Zargarian, Zeina Hobaika, Richard G. Maroun, Serge Fermandjian

**Affiliations:** 1 LBPA, UMR8113 du CNRS, Ecole Normale Supérieure de Cachan, Cedex, Cachan, France; 2 Unité de Biochimie, Département SVT, Faculté des Sciences, Université Saint-Joseph, CST-Mar Roukoz, Beyrouth, Liban; Institut Pasteur, France

## Abstract

Integration of HIV DNA into host chromosome requires a 3′-processing (3′-P) and a strand transfer (ST) reactions catalyzed by virus integrase (IN). Raltegravir (RAL), commonly used in AIDS therapy, belongs to the family of IN ST inhibitors (INSTIs) acting on IN-viral DNA complexes (intasomes). However, studies show that RAL fails to bind IN alone, but nothing has been reported on the behaviour of RAL toward free viral DNA. Here, we assessed whether free viral DNA could be a primary target for RAL, assuming that the DNA molecule is a receptor for a huge number of pharmacological agents. Optical spectroscopy, molecular dynamics and free energy calculations, showed that RAL is a tight binder of both processed and unprocessed LTR (long terminal repeat) ends. Complex formation involved mainly van der Waals forces and was enthalpy driven. Dissociation constants (Kds) revealed that RAL affinity for unbound LTRs was stronger than for bound LTRs. Moreover, Kd value for binding of RAL to LTRs and IC50 value (half concentration for inhibition) were in same range, suggesting that RAL binding to DNA and ST inhibition are correlated events. Accommodation of RAL into terminal base-pairs of unprocessed LTR is facilitated by an extensive end fraying that lowers the RAL binding energy barrier. The RAL binding entails a weak damping of fraying and correlatively of 3′-P inhibition. Noteworthy, present calculated RAL structures bound to free viral DNA resemble those found in RAL-intasome crystals, especially concerning the contacts between the fluorobenzyl group and the conserved 5′C^4^pA^3^3′ step. We propose that RAL inhibits IN, in binding first unprocessed DNA. Similarly to anticancer drug poisons acting on topoisomerases, its interaction with DNA does not alter the cut, but blocks the subsequent joining reaction. We also speculate that INSTIs having viral DNA rather IN as main target could induce less resistance.

## Introduction

Integration of the HIV-1 DNA into the host chromosome leads to the viral infection at the origin of the AIDS pandemic. Integration is catalysed by the retroviral enzyme integrase (IN) [1]–[4]. The whole integration involves the 3′-processing (3′-P) and the strand transfer (ST), which occur in the cytoplasm and in the nucleus, respectively. The integration is finalized by the cell enzymes which cleave the viral DNA 5′-overhang and fill the room left between the viral and cellular DNA [3]–[5]. A huge effort from both the public research and the pharmaceutical industry was made during the last decade to discover IN inhibitors. Only those acting on the ST step have emerged as interesting antiretroviral drugs [5]–[7]. Thus, Merck and Co has recently developed the raltegravir (RAL, MK-0518), a potent INSTI (IN ST Inhibitor) that derives from DKAs (Diketo Acids) and which is now widely used in AIDS therapy [8], [9]. However, RAL induces mutations located mainly into the loop 140 (Y143H/R/C, Q148H/R/K and G140S-Q148H) and the α4 helix (N155H) [10]–[12], entailing significant clinical resistance [11]. Positions of these mutations in the protein are consistent with the determining role hold by the α4 helix (residues 150 to 166) [13], [14] and the loop 140 (residues 140 to 149) [11], [15] of the catalytic core domain (CCD) in the IN activity and also as sites of inhibitors [15]–[18]. The crystal structures recently resolved by Hare *et al.*, 2010 confirm the coordination of two Mg^2+^ to the three coplanar oxygen or nitrogen atoms of the metal binding motif, while the halogenated aromatic ring penetrates more or less deeply into the space made available by the opening of the conserved 5′C^4^pA^3^3′ step at the end of the processed strand. The oxadiazole moiety of the RAL molecule further interacts through stacking interactions with the Tyr 143 phenolic group. However, this “π-π” stacking does not appear indispensable for the inhibitory activity of INSTIs as it can be replaced by other interactions with the loop 140 amino acid residues, as shown by the oxadiazole-lacking compounds ie EVG (elvitegravir) [15], [19], [20] and the so-called new generation of INSTIs such as MK-0536 [18], [21], MK-2048 [18], [22] and DTG (dolutegravir) [23], [24]. These compounds not only are fully active against WT-IN but they also remain effective against the RAL-resistant Y143R mutant of IN [20]. All display intermolecular contacts (“π-π”stacking) between their halogenated aromatic ring and the cytosine base of the conserved 5′C^4^pA^3^3′step. The subsequent spatial displacement of the adjacent 3′-adenine (A^3^) bearing the functional hydroxyl group from its initial position is considered as the main event promoting the inhibition of integration [15].

**Figure 1:**
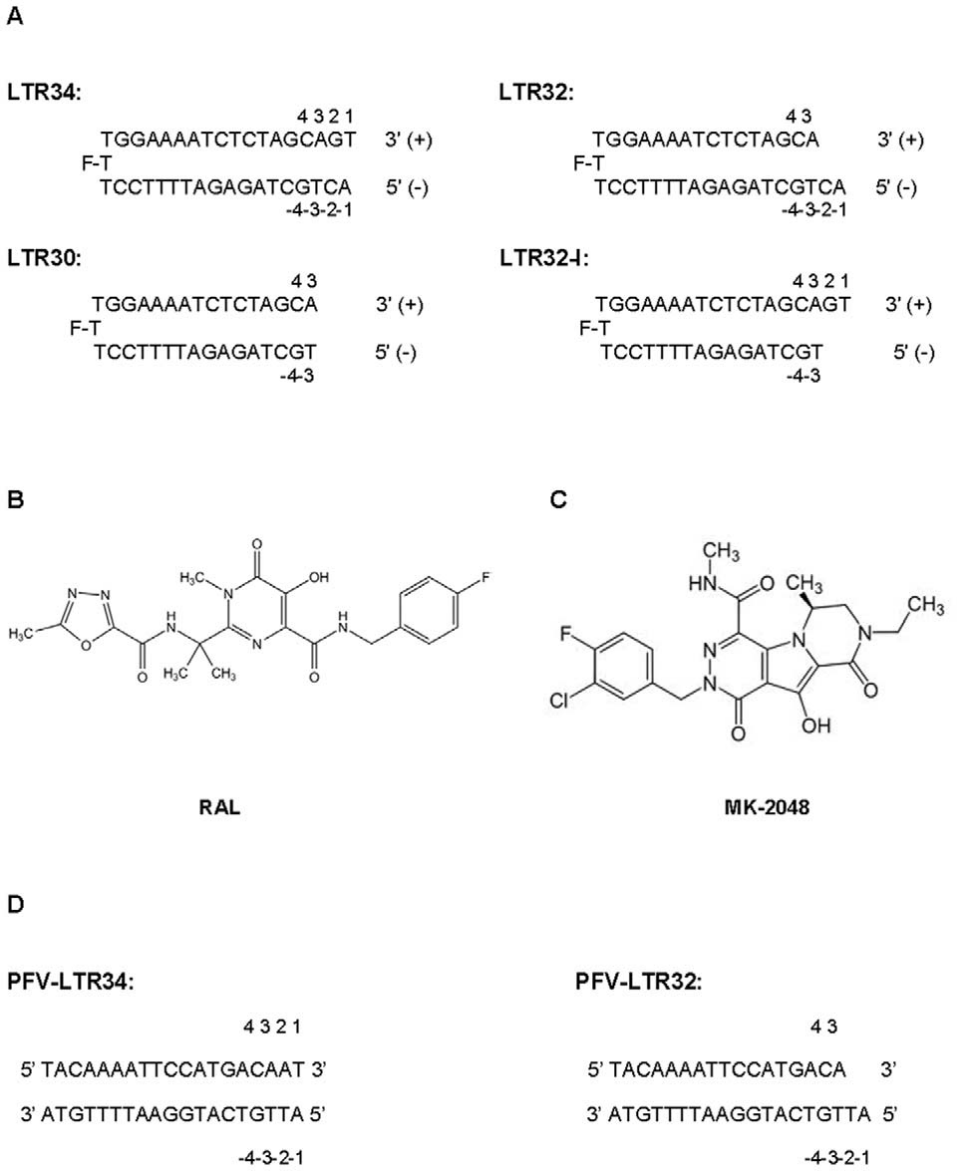
Molecules used in this study. (A) The oligonucleotides are designed to adopt a hairpin structure, folded around a three thymine loop whose central thymine bears the fluorescein reporter. LTR34: unprocessed U5-LTR end with a 17 base pair stem. The numbering of the four last base pairs (+1 to +4 and −1 to −4) starts from the ultimate 3′nucleotide on the upper (+) strand and from the ultimate 5′ nucleotide on the lower (−) strand; LTR32, processed U5-LTR, with 5′A^−1^C^−2^3′ as overhang on the (−) strand; LTR32-I, inversed LTR32, with 5′G^2^T^1^3′ as overhang in the (+) strand; and LTR30, doubly deleted LTR34 (blunt-ended DNA). (B) Chemical structure of the here studied RAL (MK-0518). (C) Chemical structure of MK-2048. This compound is given as an example of INSTI of second generation, inducing less resistance mutations in IN. (D) PFV LTR sequences used in calculations. The numbering of the four last base pairs (+1 to +4 and −1 to −4) is the same as in (A).

Actually, it is the large number of interactions occurring between INSTIs and the viral DNA, but also the inability of RAL to bind tightly to IN taken alone [25], [26], which motivated us to examine whether the LTR ends could be the primary targets of the drugs. To this end we used RAL and several oligonucleotides mimicking or deriving from the U5 LTR extremity of viral DNA (Fig. 1). Analysis of the drug-DNA complexes was performed by UV-absorbance [27], circular dichroism (CD) [27], fluorescence [13], molecular dynamics simulations (GROMACS 4.5.3/Amber99SB-ILDN) [28], [29] and free energy calculations using the Molecular Mechanics-Poisson Boltzmann Surface Area method (MMPBSA) [30]–[33]. Results indicate that one molecule of RAL binds tightly to 3′-processed LTR (Kd≈6 nM) and more weakly to unprocessed LTR (Kd≈20 nM). Binding of RAL to processed LTR requires several key nucleotides including the 5′A^−1^C^−2^3′ overhang, known for its strong implication in the binding and activity of IN [34]. The binding of RAL to this small dinucleotide strand in unprocessed LTR is facilitated by a major fraying in terminal base pairs that lowers the energy barrier for drug insertion [35], [36]. The insertion of RAL into the terminal base pairs, affects slightly their fraying and similarly the 3′-P reaction, the latter reaction being closely correlated to the motions at the LTR ends [35]. After the deletion of the 5′G^2^T^1^3′ dinucleotide, RAL blocks the ST reaction in adopting a new position at the LTR end more conducive to binding with the two divalent cations and the cytosine C^4^ of the conserved malleable C^4^pA^3^ step [15], [17], [18]. Remarkably, in our two modeled RAL-LTR32 structures, the adenine A^3^ nucleotide that bears the essential 3′OH group has conformations similar the ones found in the crystal structures of the RAL-intasome complexes (PDB codes: 3L2T [15] and 3OYA [18]). All together, present results bring greater clarity on the inhibitory mechanism of INSTIs, especially in showing that the drugs may bind specifically to both the unprocessed and processed LTR ends. The binding of RAL to unprocessed LTR does not or little impair the 3′-P reaction because the end fraying required for the cleavage of the phosphodiester backbone by IN is only weakly altered by RAL [37]. The 3′-P reaction produces a change of the complex conformation, responsible for the blocking of the joining reaction [38], [39]. We also propose that anti-AIDS drugs having an increased number of interactions with the substrate viral DNA, at detriment of the protein active site, could induce less resistance mutations.

## Materials and Methods

### Oligonucleotides and RAL

The oligonucleotides LTR34, LTR32, LTR32-I and LTR30 (Fig. 1 A) were purchased from Eurogentec (Belgium). They were designed to adopt a monomolecular hairpin structure that remains stable at the low concentrations used in fluorescence and CD experiments (10^−9^ to 10^−5^ M). LTR34 reproduces the unprocessed version of the U5 LTR end, LTR32 is the 3′-processed version obtained by deletion of G^2^T^1^3′ and LTR32-I (LTR32-inverted) is obtained by deletion of C^−2^A^−1^5′; blunt-ended LTR30 is obtained by deletion of both G^2^T^1^3′ and C^−2^A^−1^5′. The thymine at the centre of the three thymine loop bears the fluorescein reporter for fluorescence studies. Unlabeled oligonucleotides were also prepared for UV-absorbance and CD measurements. RAL (Fig. 1 B) was purchased from CacheSyn while MK-2048 (Fig. 1 C) is given as an example of an INSTI of the new generation. PFV LTR sequences used in calculations are indicated in Fig. 1D.

### UV-absorbance measurements

UV-spectrometry experiments were recorded by using an Uvikon spectrophotometer model 941 (Kontron Instruments). DNA and RAL samples were dissolved in phosphate buffer (Na/Na_2_ phosphate, 10 mM, pH 6, I = 0.05) in the presence of 5 mM MgCl_2_. Titrations were performed using the DNA as the titrant in 10 mm and 2 mm path length quartz cells and scanning the spectrum after each aliquot addition. The RAL concentration was generally maintained at 20 µM and that of DNAs was varied from 1 to 20 µM. Difference spectra between 200 nm and 380 nm were obtained by subtraction of DNA spectra from the DNA-RAL complex spectra, after subtraction of the buffer contribution.

### CD measurements

CD spectra were recorded on a Jobin-Yvon CD6 dichrograph. Measurements were calibrated with (+)-10-camphorsulfonic acid. Samples were dissolved in phosphate buffer. The concentration of oligonucleotides was 10 µM while that of the titrant was varied from 10 µM to 80 µM. Samples were placed in thermally jacketed cuvettes with 1–5 mm path lengths. Spectra, recorded with 1-nm steps and corrected for the base line, were averaged over 10 scans. Before spectral recording, samples were incubated 10 min at the chosen temperature to allow the solutions to reach their equilibrium state. Spectra of DNAs and complexes of RAL-DNA were presented as CD per residue, Δε (M^−1^.cm^−1^), as a function of wavelength, λ (nm), between 200 and 330 nm. As RAL lacks chirality it does not directly contribute to the spectrum. Yet, RAL bears several chromophores which once placed in an asymmetric environment, i.e. in the vicinity of a deoxyribose ring, can acquire chirality and generate a signal in the absorption region.

### Fluorescence measurements

These included both fluorescence intensity and anisotropy titration studies. Measurements were carried on a Jobin-Yvon Fluoromax II instrument. In fluorescence intensity titrations, RAL was maintained at a constant concentration while the DNA was used as the titrant. For RAL the wavelengths for maximum excitation and emission were λ = 313 nm and λ = 413 nm, respectively. For fluorescence anisotropy (*A* = (*I*_II_−*I_⊥_*)/(*I*_II_+2*I*_⊥_)) the parallel (*I*_II_) and perpendicular (*I*_⊥_) emission components were measured in L-format. The denominator of A was simply the total light that would be observed if no polarizers were used. With fluorescein as fluorophore grafted on DNA, the excitation from the xenon lamp (150-watt ozone-free) was performed at 488 nm with a 4-nm slit width. The emission was recorded at 516 nm with a 5-nm slit width in the case of LTR34, at 515 nm with a 4-nm slit width in cases of LTR32 and LTR32-I and at 514 nm with a 4-nm slit width in the case of LTR30. The fluorescein-labeled oligonucleotides were diluted to the desired concentration in 800 µl of phosphate buffer at the selected temperature (generally 5°C). Samples were placed in thermally jacketed 1-cm×0.5-cm quartz cuvettes, and measurements, at least 10 data points for each titration point, were recorded with an integration time of 1 s. For each fluorescence anisotropy measurement, the parallel (*I*_II_) and the perpendicular (*I*_⊥_) intensities of the background solution (*i.e.* buffer and RAL contributions) were subtracted from the sample value. The validity of fluorescence anisotropy measurements was controlled in measuring the total fluorescence intensity in parallel to fluorescence anisotropy. Variations of fluorescence intensity during these experiments were very weak, so we considered that the anisotropy signal contained the desired information on the complex formation. Kds (equilibrium dissociation constants) were calculated by fitting the sigmoidal curves, using GraphPad Prism 5 applying either the linear regression or non-linear regression (curve fit) “Least Squares” procedure. Binding stoichiometries were determined using the Bujalowsky and Lohman procedure [40]. The reverse experiment consisting in the analysis of the oligonucleotide binding to the drug was also carried out.

### Molecular dynamics simulations of viral DNA-RAL complexes

Molecular dynamics (MD) simulations were performed using the GROMACS software (version 4.5.3) [28] with the Amber99SB-ILDN force field [29]. RAL parameters were constructed using the ACPYPE (AnteChamber PYthon Parser interfacE) [41], [42], the General Amber Force Field (GAFF) [41] and the restrained electrostatic potential (RESP) charges. RESP charges were calculated using the Antechamber program [42] using the ESP charges calculated at Hartree-Fock [43] 6–31G* level [44] using G03(Gaussian03, Revision C.02). Simulations on RAL-DNA complexes were carried out on both processed and unprocessed PFV DNAs (i.e. LTR32 and LTR34) with a single Mg^2+^ ion. Initial coordinates of the LTR32-RAL complex were extracted from the 3OYA [18] PDB structure, after IN removal. Unprocessed DNA, LTR34 of PFV (Fig. 1D), was constructed by addition of the 5′AT dinucleotide (corresponding to 5′G^2^T^1^3′ in HIV) at the 3′-end of the processed strand, using the VMD program [45]. Complexes were slightly relaxed using 50 steps of steepest descent minimizer. Subsequently, the system was immersed in an explicit water box of TIP3P model [46] which extended at least 16 Å away in each direction from any DNA or RAL atom. The systems include 14993 water molecules and 35 Na ions in the case of LTR34 (46274 atoms) and 13474 water molecules and 33 Na ions in the case of LTR32 (41651 atoms).

Sodium ions were added to neutralize the system as needed for the Particle Mesh Ewald (PME) [47] calculation of the long-range electrostatic interactions, while cut-off of 10 Å was used for van der Waals (VDW) and short-range electrostatic interactions. The system was exposed to 500 step of steepest descent minimization to remove the bad contacts with the solvent. All bonds involving hydrogen atoms were constrained by LINCS algorithm [48]. Equilibration of the solvent and ions around the complexes with position constraints of the heavy atoms, were performed for two nanoseconds in the constant Number of particles, Volume, and Temperature (NVT) ensemble and in Constant Number of particles, Pressure and Temperature (NPT) thermodynamic ensemble respectively. NVT simulations were carried out using the velocity rescaling thermostat (V-rescale) [49] and the NPT using Parrinello-Rahman barostat [50] MD production simulations were performed for a total of 400 ns duration in the NPT ensemble. Moreover, we also performed a calculation of the distance (nm) evolution between the center of mass of RAL and terminal bases (5′A and 3′T) of the PFV LTR34, in the calculation course from t = 0 ns to 100 ns.

### Binding free energy calculations

RAL binding free energies were estimated using the end point Molecular Mechanics Poisson-Boltzmann Surface Area (MMPBSA) method [30]–[33], [51]. The total free energy (G) in MMPBSA analysis for a given species (complex, receptor, and ligand) was determined using Eq. (1); the overall change in free energy for complex formation (ΔG_bind_) for a non-covalent binding event was calculated according to Eq. (2).
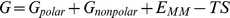
(1)

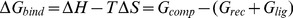
(2)The polar solvation energies (G_polar_) were computed in continuum solvent using Poisson-Boltzmann (PB) and ionic force of 0.05 as used in the experiments. The non-polar terms (G_nonpolar_+γSASA+β) were estimated using solvent accessible surface areas (SASA in Å [29]) with typical values for γ = 0.00542 kcal/mol Å^2^ [29] and β = 0.92 kcal/mol. The E_MM_ term represented the sum of the electrostatic (Coulombic), VDW (Lennard-Jones), and internal energies (bonds, angles, and dihedrals). The remaining term represented temperature (T) and solute entropy (S), which can be estimated from normal-mode analysis of energy-minimized structures or quasi-harmonic (QH) modes over stabilized region of MD trajectory. 500 snapshots were selected from the last 5 ns, by keeping the snapshots every 5 ps. The entropy contributions were estimated by QH mode using 5000 frames from the last 5 ns. The free energy analysis was carried out using MMPBSA.py script from Amber11 program.

## Results and Discussion

DNA is a target for a wide diversity of ligands. Due to the complexity and variety of DNA structures, the binding modes of these ligands, including anticancer agents, are them also complex and varied (intercalation, groove binding, insertion in breaks…) [27], [38], [39], [52]–[54]. Often, a same molecule (for instance ellipticine) can act as both an intercalator and a groove binder [27]. Moreover, anticancer agents such as camptothecin and derivatives, poisons of topoisomerase I, operate through insertion into a break of the DNA double helix created by the enzyme in one DNA strand [55]. Most of results stipulate that base pairs, double helix grooves and strand breaks in DNAs can be primary binding sites for topoisomerases inhibiting drugs [39]. As IN, similarly to topoisomerases, is also a DNA cutting and joining enzyme, we decided to investigate the binding of its best known inhibitor, RAL, to DNA. To this end, we used UV absorbance, CD, fluorescence and molecular dynamics with oligonucleotides to assess the binding of the drug to viral DNA LTR ends with respect to its INSTI activity.

### UV-absorbance measurements

**Figure 2:**
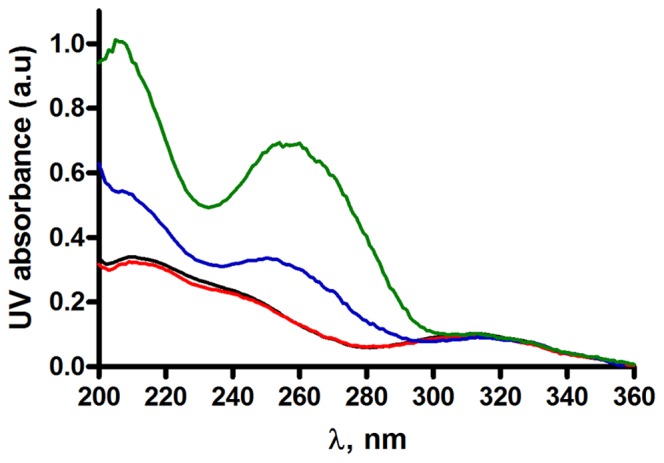
UV-absorption analysis of oligonucleotides. Spectra of RAL 20 µM (black) together with 20 µM LTR32 (green), 20 µM LTR34 (blue), 20 µM LTR30 (red), in phosphate buffer pH 6, I = 0.05, and MgCl_2_ 5 mM final concentration.

The first evidence of an interaction of RAL with the terminal part of viral DNA either unprocessed or processed is provided by UV-absorption titrations (Fig. 2 and Fig. S1 A and B). UV spectra of selected oligonucleotides, LTR34, LTR32 and LTR30 (Fig. 1 A), between 200 and 380 nm, display a main signal centred at about 260 nm and an additional peak around 200 nm, characteristics of B-DNA (Fig. S1 A). The UV spectrum of RAL (Fig. 1 B) between 200 nm and 380 nm consists in two peaks at 210 nm and 313 nm and a shoulder at 245 nm (Fig. S1 A), corresponding to the contributions of the aromatic chromophores making up the molecule. Fig. 2, shows the variations of the RAL spectrum resulting from addition of 20 µM LTR32, LTR34 and LTR30 to 20 µM RAL (stoichiometry 1∶1) after subtracting the spectrum of free DNA at the same concentration. Completely different effects are observed. With addition of LTR32 there is an emergence of a signal at about 260 nm, exactly where the DNA contributes, consistent with a change of conformation in LTR32 in response to drug-DNA complex formation (Fig. S1 B). The increase of intensity of the band at ≈205–210 nm, could arise from changes in the contributions of both RAL and DNA, due to their interaction. In contrast, the broad signal of RAL centred at 313 nm did not manifest any change, suggesting that the RAL chromophore generating this signal remains free of interactions in the complex. Noteworthy, addition of unprocessed LTR34 to RAL produces the same effects as shown by LTR32, but, however, less intense, while addition of blunt ended LTR30 is without effects. Taken together the UV-absorption results shows that RAL binds to the terminal part of LTRs, with a preference for the 3′ processed DNA that carries the 5′A^−1^C^−2^3′dinucleotide overhang. In unprocessed LTR34 the 5′A^−1^C^−2^3′ dinucleotide is involved in a duplex with the undeleted 5′ G^2^T^1^3′ dinucleotide, but this does not constitute a rigid barrier to the binding of RAL. This can be explained by the important end fraying in unprocessed LTR [35], [36] that facilitates the RAL accommodation into the terminal base pairs. The inability of RAL to bind the blunt ended oligonucleotide LTR30, which is further devoid of both IN binding and ST activities [34], confirms the functional importance of the terminal 5′A^−1^C^−2^3′ step within either the unprocessed or the processed viral DNA, regarding the capture of ligands.

### CD spectroscopy measurements

**Figure 3:**
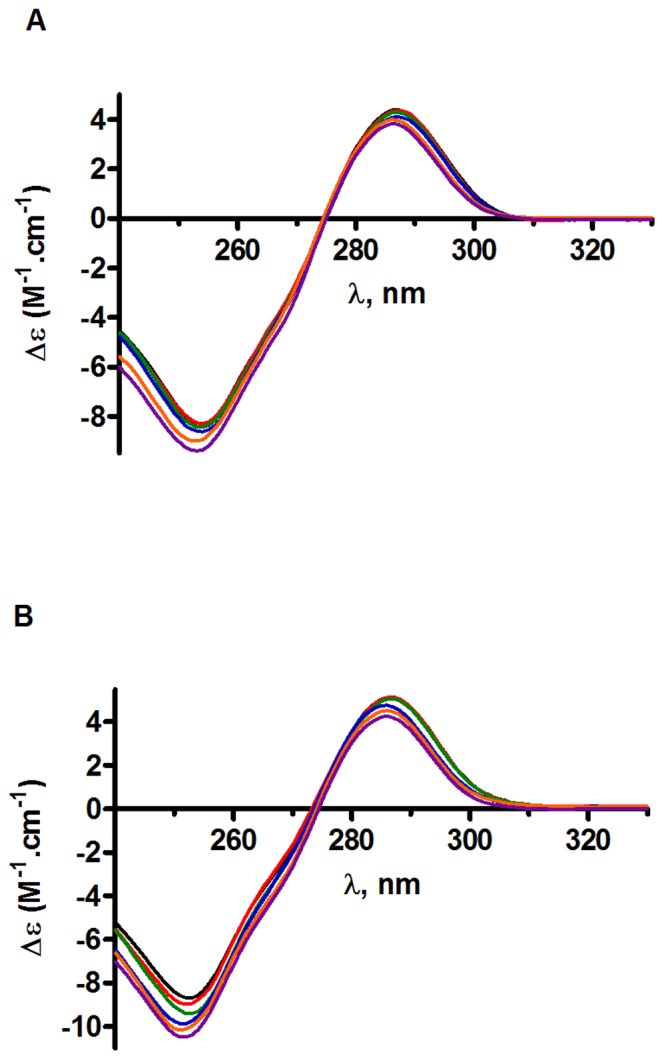
Circular dichroism analysis of oligonucleotides-drug complexes. Spectra of LTR34 (A) and LTR32 (B) at 10 µM (black) and difference spectra [LTR32/34 (10 µM)+RAL (10 µM, red; 20 µM, green; 40 µM, blue; 60 µM, orange; and 80 µM, purple)−LTR32/34 (10 µM)], in phosphate buffer pH 6, I = 0.05, and MgCl_2_ 5 mM final concentration.

The CD technique is widely used to determine the secondary structures of proteins and nucleic acids and to follow the conformational changes induced by their mutual association or their binding to any type of ligand [56], [57]. Here, CD was applied to study the binding of RAL to LTR34 and LTR32, which mimic the unprocessed and processed viral U5 DNA ends, respectively (Fig. 1 A). The spectra of LTR34 and LTR32 presented in Fig. 3 show two main signals (negative at ≈250 nm and positive at ≈280 nm) characteristics of B-DNA. In titration experiments, RAL was added (concentrations from 10 µM to 80 µm) to oligonucleotides maintained at 10 µM concentration. The spectra recorded at drug: DNA ratios of 1, 2, 4, 6 and 8 showed a gradual variation of intensity of the two B-DNA signals at ≈250 nm and ≈280 nm. Similarly to UV-absorption experiments, effects were larger with LTR32 than LTR34. However, the signals were not shifted and no new signal induced by drug chromophores buried in a chiral environment was detected. The CD changes were assigned to a rearrangement of the nucleotide bases at the RAL binding site or contiguous to it [27], [58]. We will subsequently observe that it is a single RAL molecule that is inserted at the LTR extremity, so that one cannot expect the generation of a large signal.

### Fluorescence measurements

The quantification of interactions stabilizing partner molecules is essential to the understanding of the complex formation. Fluorescence intensity and anisotropy measurements are well suited to a quantitative analysis of complexes as long as one of the binding partners is fluorescent. The fluorescence anisotropy gives information on both the stoichiometry of the complex and the binding constant: Kd = [L]×[R]/[LR] (Kd: dissociation constant; L: ligand and R: receptor) [13], [26], [59], [60]. The Kds provided by fluorescence anisotropy are confirmed by fluorescence intensity experiments, where the fluorescent ligand molecule is titrated with increasing concentrations of oligonucleotides.

**Figure 4:**
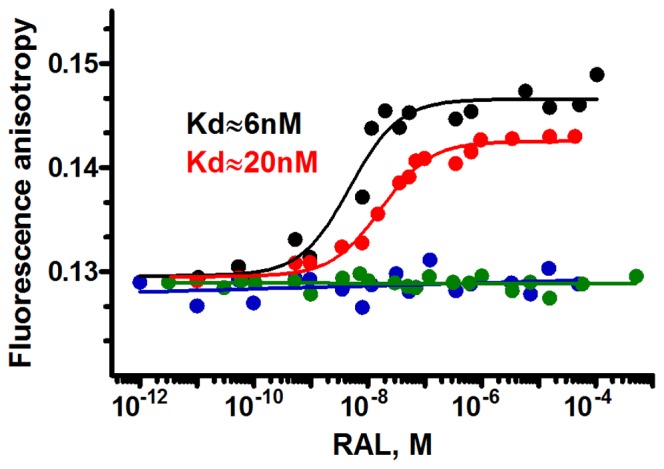
Quantitative analysis of RAL binding to oligonucleotides. Fluorescence anisotropy titration of the four oligonucleotides LTR32 (black), LTR34 (red), LTR32-I (blue) and LTR30 (green) at 20 nM by increasing concentrations of RAL (from 10^−12^ M to 10^−4^ M). Kds obtained from titrations of LTR32 and LTR34 at 20 nM are indicated near the corresponding curves.

**Figure 5:**
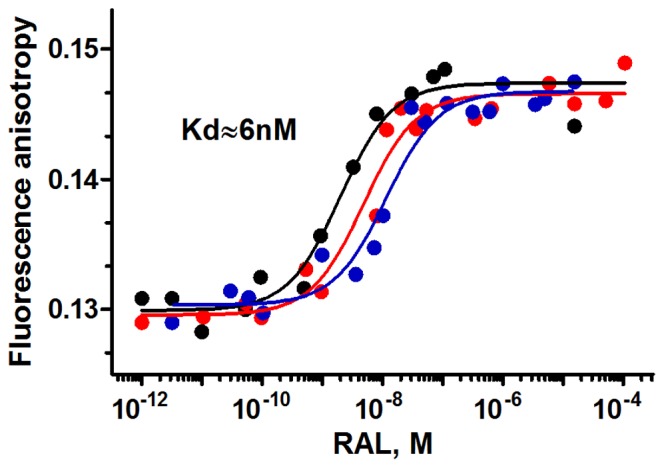
Thermodynamic parameters for the binding of RAL to LTR32. Titration of LTR32, at three different concentrations: 9 nM (black), 20 nM (red), and 30 nM (blue). Curve treatment provided a 1∶1 stoichiometry for the complex formation and an average Kd of ≈6 nM for the binding affinity. Samples were in phosphate buffer pH 6, I = 0.05, at 5°C, MgCl_2_ 5 mM final concentration.

The equilibrium saturation curves of the four fluorescein labeled oligonucleotides and increasing concentrations of RAL recorded by fluorescent anisotropy are shown in Fig. 4 and 5. The results reported in Fig. 4 confirm the above UV-absorbance and CD experiments, which indicated that RAL was able to interact with processed LTR32 and unprocessed LTR34, but fail to interact with the 3′ dangling ended LTR32-I and the blunt ended LTR30. Kds determined at midpoints are of ≈6 nM for LTR32 and ≈20 nM for LTR34. Rather similar values were obtained with the fluorescence intensity approach, using the oligonucleotides to titrate RAL (Fig. S2). The stoichiometry for the binding of RAL to LTR32 was determined by titrations at three different DNA concentrations (9 nM, 20 nM and 30 nM) (Fig. 5). The monophasic curves reached a same plateau after a variation of anisotropy of ΔA≈0.015. The three Kd values were quite similar and provided a mean value of ≈6 nM. Application of the Bujalwski and Lohman procedure [40], showed that a single RAL molecule was bound to LTR32 (1∶1 stoichiometry), in agreement with the crystal structure results [15]. Noteworthy, the experimental Kd for the binding of RAL to intasome [61] is higher the one found for LTR32 alone (19 nM vs 6 nM).

The above results have several implications. First, the fact that RAL binds to LTR34, is consistent with an end fraying lowering the energy barrier for RAL accommodation into the terminal bases. In retroviral DNAs, the fraying of terminal base pairs seems amplified by the small 5′C^4^A^3^∶3′G^−4^T^−3^ duplex just before in the sequence. This dinucleotide duplex, invariant in retroviral DNAs, is considered as one of the less stacked and most malleable dinucleotide [62], [63]. It could contribute to increase the motions and disruption of the connected base pairs [35], [36], [62], [63], which according to several authors facilitate the IN binding and the scissile bond cleavage [35], [36]. Present study shows further that the end fraying in LTR may contributes to the capture of INSTIs, but the latter has no significant impact on the cut of the scissile bond, as INSTIs are generally weak 3′-P inhibitors. Second, the ability of RAL to bind both LTR32 and LTR34 and its inability to bind both the blunt ended LTR30 and the 3′ dangling ended LTR32-I, suggests that the terminal 5′A^−1^C^−2^3′ dinucleotide is required for the drug-DNA complex formation, either as a dinucleotide overhang at the end of processed LTR or as a dinucleotide within a duplex subject to a large fraying at the end of unprocessed LTR. Actually, a fair amount of data has been reported on the stabilizing role of the 5′A^−1^C^−2^3′ overhang in the complex of IN with LTR [34], [64]. Here, we understand that the overhang can be also involved in the LTR-RAL complex stability.

### Molecular modelling

Our UV-absorption, CD and fluorescence experiments provide insight on the binding of RAL to the processed (LTR32) and unprocessed (LTR34) DNAs. However, they do not give information on the binding events and the type of interactions stabilizing molecular complexes. On the other hand, MD simulations can provide atomic details on structural and dynamic events governing the complex formation, while MMPBSA provides the binding free energies and allows the quantification of the complex stability [30], [51], [65]. We performed MD simulations (400 ns in total) and MMPBSA calculations in order to unravel the role of viral DNA ends as possible primary targets in the mechanism of IN inhibition by RAL. Indeed, MMPBSA overestimate the binding values, which is not surprising since this method is known for this defect [51], [66]. However, the MMPBSA values presented hereafter provide the same ranking as that given by fluorescence, in showing that the complexes of RAL with processed LTR are more stable than those with unprocessed LTR.

### Equilibration of the MD simulations

**Figure 6:**
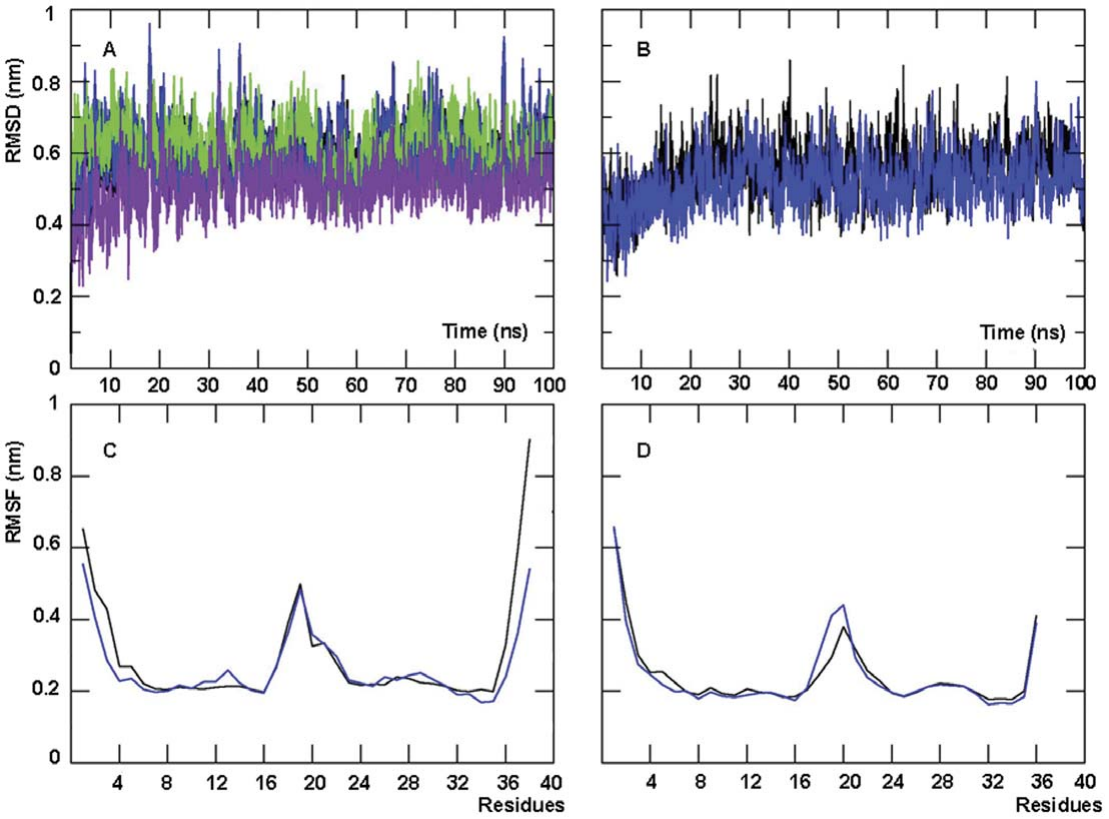
MD simulations of the RAL-LTR34 and RAL-LTR32 complex systems (PFV oligonucleotides), using GROMACS with the AMBER force field. (A) Time evolution of RMSD (root mean square deviation) values based on all the heavy atoms for the two LTR34 trajectories (black: LTR34-1 and blue: LTR34-2). RMSD calculations for a single trajectory were also performed using the sugar C4′ atoms (green: LTR34-1) and repeated for LTR34 devoid of 3′-AT (purple). (B) Time evolution of RMSD values of LTR32 for two trajectories (black: LTR32-1 and blue: LTR32-2). (C) RMSF (root mean square fluctuation) variations of sugar C4′ atoms for LTR34 and (D) RMSF variations of sugar C4′ atoms for LTR32.

We applied MD simulations to the analysis of RAL complexes with LTR34 and LTR32 ends. MD trajectories monitored by the root-mean-square displacement (RMSD) values of heavy atoms with respect to the X-ray structure (PDB code: 3OYA) are shown in Fig. 6A and B. Similar RMSD values were obtained when the sugar C4′ atoms (green curve in Figure 6A for LTR34) or the phosphorus atoms (not shown) were monitored. The trajectories show that the system is stabilized after 2 ns and conserves the same RMSD value till the end of the simulation (100 ns) for both LTR34 and LTR32. A duplicate simulation also of 100 ns gives similar results. LTR34 displays higher RMSD values than LTR32 (the purple curve in Fig. 6A is very similar to the black and the blue in Fig. 6B). The important fraying (base pair disruption or impairing) of the two terminal base pairs of LTR34, is the main reason of the greater flexibility of LTR34 in comparison with LTR32. This is particularly obvious in the root-mean-square fluctuation (RMSF) of the sugar C4′ atoms (see Fig. 6C and D).

### RAL binding to DNA

**Figure 7:**
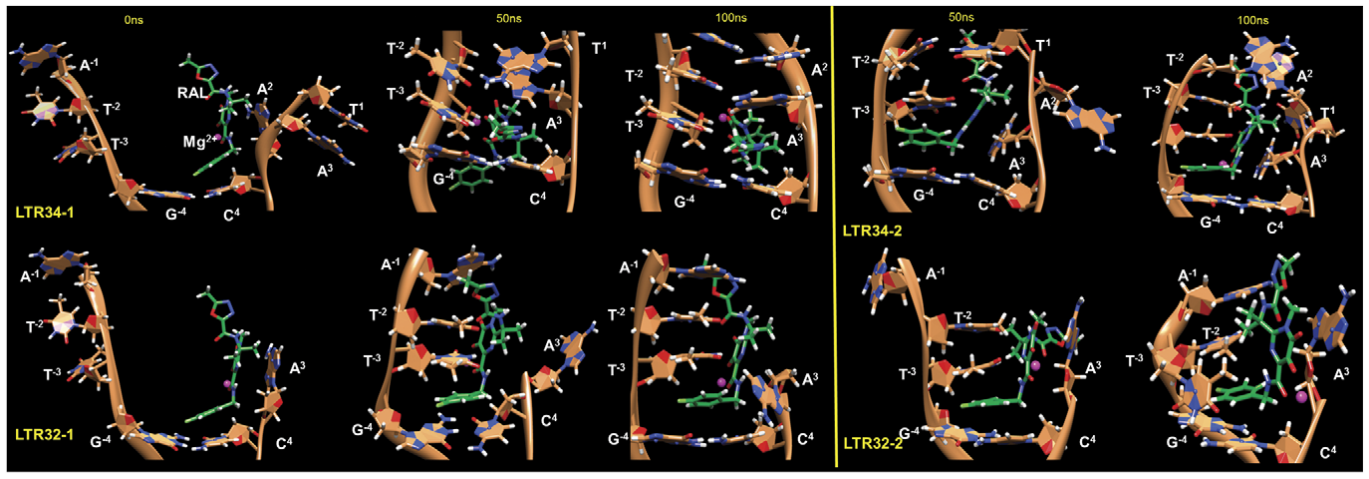
Snapshots from the two 100 ns trajectories of RAL in complex with unprocessed LTR (LTR34-1 and 2, top) and processed LTR (LTR32-1 and 2, bottom). RAL is colored in slime green and bases in sandy brown, except for atoms at interacting distances which are colored using the usual code (hydrogen in white; nitrogen in blue; and oxygen in red) except for carbons, while the Mg^2+^ ion is represented by magenta ball. Selected snapshots are 0 ns (the initial structure), 50 ns and 100 ns.

Each oligonucleotide yields two complexes: RAL-LTR34-1, RAL-LTR34-2 and RAL-LTR32-1, RAL-LTR32-2 (Fig. 7). Table 1 summarizes the free energy values for the binding of RAL to the LTR34 and LTR32 ends, calculated with the MMPBSA method. Values agree with a favorable binding of RAL to both unprocessed LTR34 (RAL-LTR34-1, RAL-LTR34-2) and processed LTR32 (RAL-LTR32-1, RAL-LTR32-2). The binding energy characterizing RAL-LTR34-1 is less favorable compared with RAL-LTR34-2, and also compared with the two other complexes, RAL-LTR32-1and LTR32-2. Compared with RAL-LTR34-2 (and the other two complexes), RAL-LTR34-1 displays a very distinct binding mode of RAL. In fact, it is the only case where the drug uses its oxadiazole moiety to bind DNA. The ring intercalates in between the terminal base pairs, while the remaining of the molecule is solvent exposed. In RAL-LTR34-2, RAL uses its fluorobenzyl moiety to interact with the G^−4^ and the T^−3^ bases, and its pyrimidine ring to interact with the A^3^ of the conserved C^4^pA^3^step. Interactions of the fluorobenzyl moiety with of G^−4^ and T^−3^ bases are also found in both RAL-LTR32-1 and RAL-LTR32-2. In RAL-LTR32-1 the fluorobenzyl group interacts with the C^4^ base, while in RAL-LTR32-2 the pyrimidine ring interacts with the A^3^ base.

**Table 1:** Calculated binding parameters for the complexes of RAL with LTR32 and LTR34.

Complex	Δ E_ele_	Δ E_VDW_	Δ E_MM_	Δ G_PB_	Δ G_SASA_	Δ H	TΔS	Δ G
LTR34-1	258.9±17.0	−30.3±4.0	228.6±15.0	−256.1±15.0	−2.4±0.2	−29.9±3.7	−18.7	−11.2±3.7
LTR34-2	253.0±13.0	−48.5±4.0	204.5±12.5	−249.2±12.7	−3.3±0.2	−48.0±5.0	−16.6	−31.4±5.0
LTR32-1	194.3±19.7	−36.8±3.5	157.5±18.9	−200.8±18.5	−3.0±0.1	−46.3±4.0	−15.1	−31.2±4.0
LTR32-2	166.6±11.0	−35.0±4.0	131.6±10.7	−171.5±10.3	−3.1±0.2	−43.0±5.3	−17.5	−25.5±5.3

The free energy ΔG_MMPBSA_ from two trajectories for each system (LTR34-1, 2 and LTR32-1, 2) and averaged over 500 frames from each trajectory. Energies and standard deviations are given in kcal/mol. E_ele_: Coulombic energy; E_vdw_: van der Waals energy; E_MM_: total molecular mechanics energy (E_ele_+E_vdw_); G_PB:_ polar solvation free energy based on Poisson-Boltzmann; G_SASA_: Non-polar solvations free energy based on SASA; TΔS: the entropy contribution to the binding calculated by the QH; ΔG: the total free energy.

**Figure 8:**
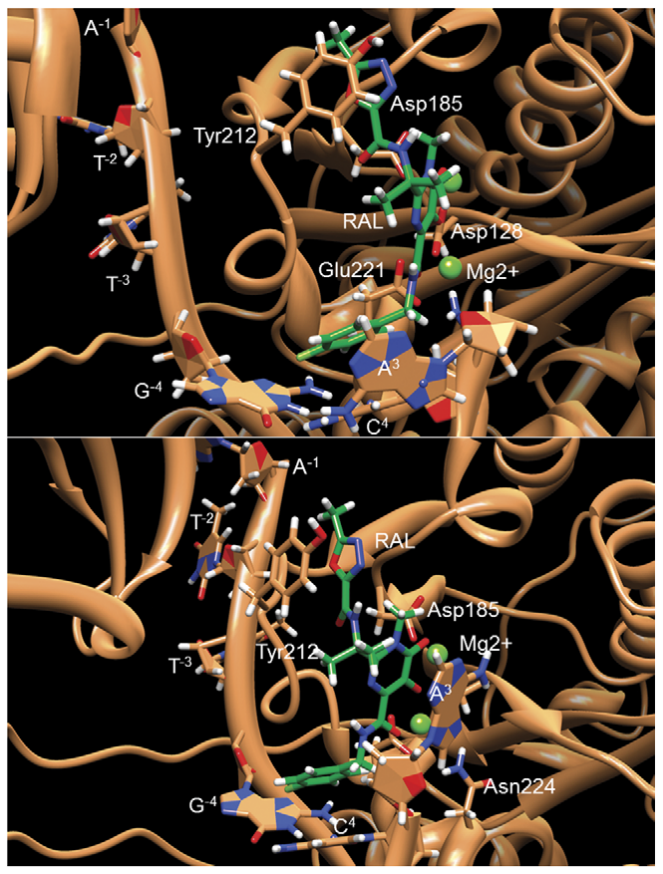
Details of the interactions of RAL with its surrounding amino acids and nucleotides as observed in the two X-ray structures of RAL bound to the PFV intasome (pdb codes: 3L2T and 3OYA). RAL is shown in slime green and IN and LTR residues in sandy brown. The amino acids and nucleotides giving interactions are shown in sticks with hydrogens in white, nitrogens in blue and oxygens in red. Mg^2+^ ions are represented by light green balls. The main difference between 3L2T and 3OYA structures concerns the orientation of the adenine A3.

It is worth noting, that the terminal adenine A^3^ bearing the recessed 3′-hydroxyl, samples two different conformations in the calculated RAL-LTR32 complexes. These two conformations are found in the crystal structures of RAL bound to the PFV intasome (PDB codes: 3L2T [15] and 3OYA [18]). In the corresponding Fig. 8, the role of the fluorobenzyl ring containing moiety appears essential to stabilization of the RAL-DNA complex and consequently to induction of inhibition. In three complexes out of four provided by calculations, the ring partakes in key interactions. Actually, the stacking of the aromatic ring on the cytosine C^4^ base and the interactions of the RAL amide group with the adenine A^3^ sugar in the invariant 5′C^4^A^3^3′step, as well as the interaction of the fluorine atom with the guanine G^−4^ at the back of the cavity, are all found in the crystal structures of INSTI-intasome [18]. Moreover, the fluorobenzyl moiety in RAL-LTR34-2 displays the same stabilizing interactions than in RAL-LTR32-1, or 2, and in the crystal structures of RAL-intasome [15].

### Complex stabilities

The calculated binding energies are shown in table 1. We note that when the fluorobenzyl moiety is not involved in interactions (RAL-LTR34-1), the binding energy is the highest, which confirms the key role of the halogenated moiety in the complex stabilization. In the four RAL-DNA complexes, the van der Waals (VDW) interactions (E_vdw_) are predominating which is a relatively common feature in complexes of organic ligands with nucleic acids [67]. In the two complexes of RAL with unprocessed LTR34, the fluorobenzyl ring has much more favorable VDW interactions compared with the oxadiazole group: ([LTR34-2]_vdw_−[LTR34-1]_vdw_ = −18 kcal/mol). The solvent (ΔG_PB_+ΔG_SASA_) also contributes to a favorable binding, while both the electrostatics and the entropy are not favorable to the binding. The latter affects apparently equally the binding of RAL to LTR34 and to LTR32. Since the electrostatics and the solvation forces neutralize each other, the VDW interactions become the main binding contribution. Finally, the binding of RAL to viral DNA ends appears as an enthalpy driven process, rather than an entropy driven one.

### Binding free energies: comparisons of simulated with experimental structures

The free energy, ΔG, values for the binding of RAL to LTR34 and LTR32, provided by the MMPBSA methodology are overestimated compared with the experimental ΔG values determined by fluorescence anisotropy (ΔG_LTR32_≈−10.5 Kcal/mol and ΔG_LTR34_≈−9.8 Kcal/mol). This result is not unexpected as MMPBSA is known for this defect, especially when a non-polarizable force field is used. Yet, the fluorescence titration experiments and the molecular dynamics approaches lead to similar conclusions. Both show that RAL can form stable complexes with LTR34 and LTR32. The MMPBSA calculations predict a binding of RAL to viral DNA which is enthalpy driven and assign an important role to the VDW forces in the complex stabilization. The finding of an enthalpy driven binding is not so surprising, as RAL, similarly to intercalators, also used π-π stackings to interact with the bases of DNA (Fig. 7). A dataset consisting of 26 binding interactions has shown that intercalating molecules bind to DNA with a favorable enthalpy contribution [68], while the binding of groove-binders is due more to a favorable entropy [68]. Actually, the binding of RAL to LTRs is characterized by a mean ΔH/ΔG ratio of about 1.50, while a ΔH/ΔG ratio ranging from 0.83 to 1.97 is considered as a clear signature of enthalpy driven binding [68]. Although RAL has a structure reminiscent of some intercalators, our hydrodynamic studies (not shown) indicate that it is unable to insert into base pairs of the DNA double helix. Indeed, the anchoring of RAL at the end of unprocessed LTR needs of the end fraying. In processed LTR the room opened at the DNA end by the release of the 5′G^2^T^1^3′ (5′A^2^T^1^3′ for IN-PFV) dinucleotide facilitates the RAL interaction. In both processed and unprocessed LTRs we find that the 5′A^−1^C^−2^3′ (5′A^−1^T^−2^3′ for PFV IN) dinucleotide partakes in the DNA-drug complex stabilization. Yet, in the crystal structure of the ternary complex of RAL with the PFV intasome, the 5′AT3′ overhang rather interacts with the protein [15] as it was already the case in the intasome [15]. Here, either the interactions of the 5′A^−1^T^−2^3′ overhang could be stronger with the protein than with RAL, and be prioritized, or the crystal packing could prevent RAL from reaching its target. Noteworthy, RAL makes van der Waals contacts with both the invariant 5′C^4^pA^3^3′step known for its functional importance at the LTR end [34], [61] and the guanine G^−4^ facing the cytosine C^4^ of this step. These interactions are also found in calculated and crystal structures of DTG, a structural analogue showing activity against RAL-resistant mutants [69]. In that case it has been suggested that DNA could make the greatest energetic contribution to DTG binding.

The particular properties of LTR ends contribute to the molecular fitting of RAL to DNA prior 3′-P. Owing to the high flexibility prevailing at the unprocessed DNA end the energy loss during complex formation is weak. This is especially true with regard to the drug intercalation which is an entropy costly mechanism requiring both base pair destacking and DNA distortion to create a suited site for the binding, and resulting in a damping of motions and stiffening of the duplex.

### Conclusion

**Figure 9:**
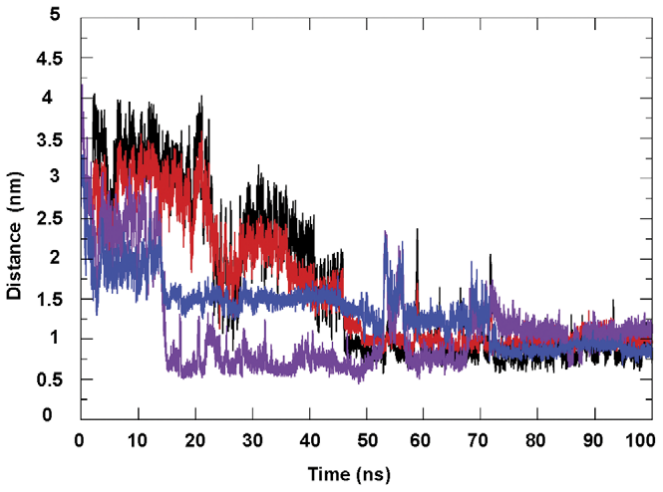
Effects of RAL on the distance and the fraying of unprocessed LTR ends. Time evolution of the distance (nm) between the mass of RAL (D) and that of the terminal bases (T and A) of LTR34-1 (black:D-T and red: D-A) and LTR34-2 (purple: D-T and blue: D-A). Interaction of RAL with the terminal bases decreases the distance between the drug and the bases and also reduces moderately the end fraying.

Our experimental and theoretical studies underline the particular role of the LTR terminal nucleotides in the binding of RAL to viral DNA. In previous reports, the antiviral drug RAL has been described as an INSTI acting at the interface of DNA-enzyme according to an important lattice of interactions with the processed LTR end, two metal ions and IN [3], [15], [18]. The recently published X-ray crystallography results describing the binding of INSTIs to the PFV-intasome, have confirmed most of the previous biochemical observations, including the two metal binding, and provided outstanding information on the inhibition mechanism used by inhibitors [15], [17], [18]. Yet, so far reported studies had not addressed the possible binding of IN inhibitors to the viral DNA end, especially prior to 3′-P. Actually, like other small organic molecules with chemotherapeutic activities, RAL binds directly and selectively to DNA. However, in contrast to the anticancer agents, as for example anthracyclines and ellipticines, RAL occupies selective binding sites on DNA. Its binding to the unprocessed LTR end could be at the basis of the observed 3′-P inhibition. What appears is that the capture of the drug by unprocessed LTR entails a small damping of motions (fraying) in the terminal base pairs (Fig. 9). Initial studies [35], [36], [70]–[73] have shown that any restriction of fraying in the terminal base pairs by either extension of the duplex [35], [71] or chemical linkage of the duplex ends [73] impairs the 3′-P reaction. Remarkably, RAL keeps the same conformation within the complex with unprocessed and processed DNAs, with its halogenated ring in face to face contact with the cytosine base of the conserved 5′C^4^pA^3^3′ step, and the adenine A^3^ bearing the recessed 3′-hydroxyl group moved from its operative position.

At the end our work could help to better understand some of the factors contributing to the mechanism of action of IN inhibitors. Above all, we have shown that RAL binds to the LTR end prior 3′-P reaction, but the impact on this reaction is small. The fact that the contacts established by RAL with the processed LTR end mainly concern the nucleotides C^4^, A^3^ and G^−4^, pushes us to speculate that new drugs giving an increased number of interactions with these highly conserved bases, at expense of interactions with amino acid side chains of the protein active site, will be better INSTIs and weaker resistance inducers.

## Supporting Information

Figure S1 **UV-absorption analysis of oligonucleotides and raltegravir free and in complexes.** (A) Spectra of RAL (80 µM, in red) and LTR32 (10 µM, in black) in phosphate buffer pH 6, I = 0.05, MgCl_2_ 5 mM final concentration. (B) Spectra of RAL 20 µM, (black), in complex with LTR32 (1 µM, red), LTR32 (5 µM, blue) and LTR32 (10 µM, green).
(TIF)

Figure S2 **Binding of oligonucleotides to raltegravir.** Titration data for LTR32 (black) and LTR34 (red) and corresponding Kds are obtained from fluorescence intensity in so called reverse experiments. The spectra of raltegravir recorded at different LTR34 concentrations are given in insert.
(TIF)
