# Supplementary figures and images for: Unprocessed Viral DNA Could Be the Primary Target of the HIV-1 Integrase Inhibitor Raltegravir
Source: PLoS One. 2012 Jul 2;7(7):e40223. doi: 10.1371/journal.pone.0040223 (PMC3388078; doi:10.1371/journal.pone.0040223)

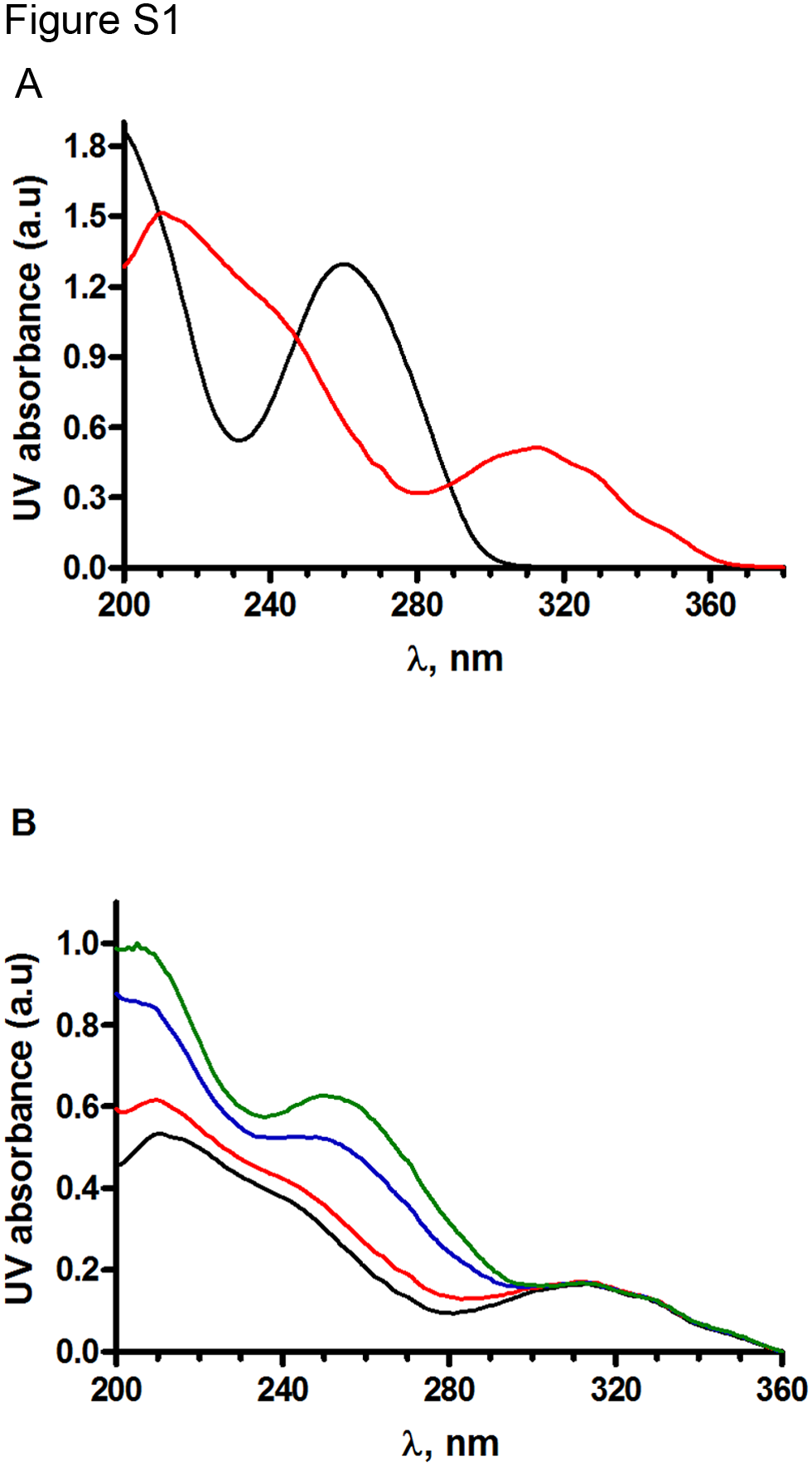

Supplement: Figure S1 — UV-absorption analysis of oligonucleotides and raltegravir free and in complexes. (A) Spectra of RAL (80 µM, in red) and LTR32 (10 µM, in black) in phosphate buffer pH 6, I = 0.05, MgCl2 5 mM final concentration. (B) Spectra of RAL 20 µM, (black), in complex with LTR32 (1 µM, red), LTR32 (5 µM, blue) and LTR32 (10 µM, green). (TIF) [file pone.0040223.s001.tif]

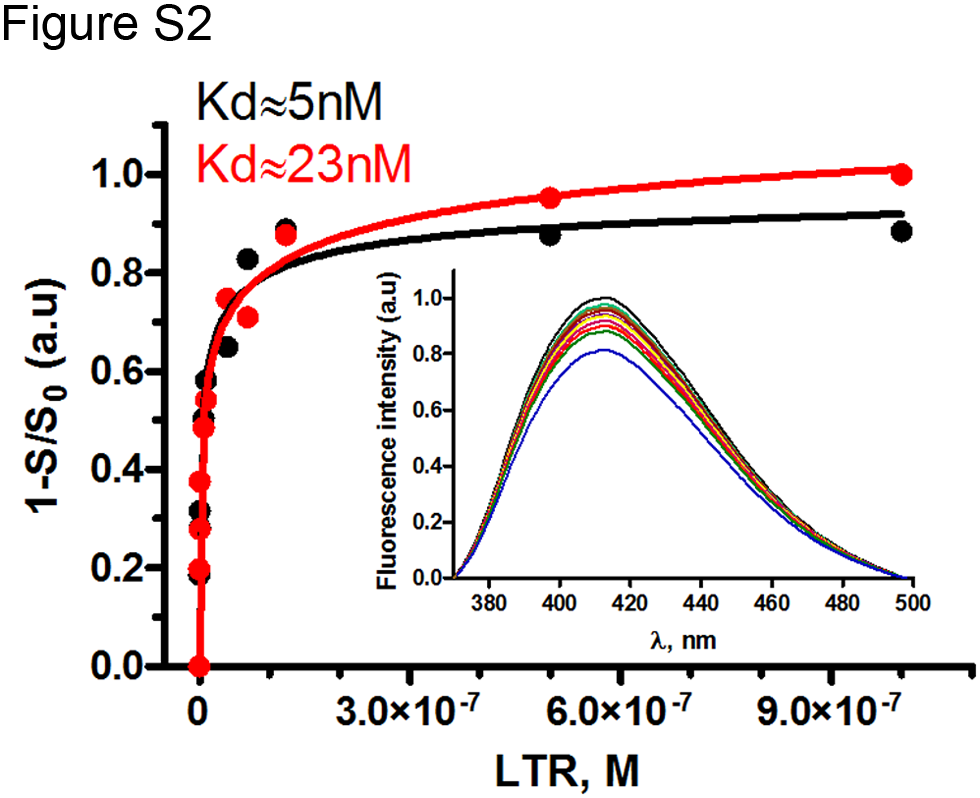

Supplement: Figure S2 — Binding of oligonucleotides to raltegravir. Titration data for LTR32 (black) and LTR34 (red) and corresponding Kds are obtained from fluorescence intensity in so called reverse experiments. The spectra of raltegravir recorded at different LTR34 concentrations are given in insert. (TIF) [file pone.0040223.s002.tif]
